# A Novel Technique of Arthroscopic Femoral Tunnel Placement during Medial Patellofemoral Ligament Reconstruction for Recurrent Patellar Dislocation

**DOI:** 10.3390/jcm12020680

**Published:** 2023-01-15

**Authors:** Fengyi Hu, Weili Shi, Haijun Wang, Cheng Wang

**Affiliations:** Department of Sports Medicine, Peking University Third Hospital, Institute of Sports Medicine of Peking University, Beijing Key Laboratory of Sports Injuries, Beijing 100191, China

**Keywords:** medial patellofemoral ligament reconstruction, femoral tunnel, arthroscopy, anatomic landmarks

## Abstract

Recurrent patellar dislocation is a commonly encountered patellofemoral disease. Prompt surgical intervention is indicated for recurrent dislocation to restore patellofemoral stability. As one of the most preferred procedures, medial patellofemoral ligament (MPFL) reconstruction has been implemented on a large scale. Femoral tunnel placement remains a crucial technical issue during MPFL reconstruction and is critical to ensure the isometry and proper tension of the graft. Currently, visual–palpatory anatomic landmarks and fluoroscopy-guided radiographic landmarks comprise the main approaches to intraoperative femoral tunnel positioning. However, the accuracy of both methods has been questioned. This article introduces an arthroscopic femoral tunnel placement technique. Apart from traditional anteromedial and anterolateral portals, two auxiliary arthroscopic portals are specially designed. The adductor tubercle, the medial epicondyle and the posterior edge are selected as main anatomic landmarks and are directly visualized in sequence under arthroscope. The relative position between the femoral attachment of the MPFL and the three landmarks is measured on preoperative three-dimensional computed tomography, providing semi-quantified reference for intraoperative localization. This technique achieves minimally invasive tunnel placement without X-ray exposure, and especially suits obese patients for whom palpatory methods are difficult to perform.

## 1. Introduction

Recurrent patellar dislocation is a patellofemoral disease particularly common in young active patients [1]. Repeated dislocations usually lead to injury of the medial patellar soft-tissue stabilizers and irreversible chondral lesions, which subsequently cause chronic instability and patellofemoral pain [2]. Therefore, surgical interventions are widely recommended in the treatment of recurrent patellar dislocation [3]. Currently, medial patellofemoral ligament (MPFL) reconstruction is the mainstream method among multiple procedures reported in the literature [4].

The MPFL starts from the “saddle sulcus” between the adductor tubercle (AT) and the medial epicondyle (ME) with attachment to the superomedial aspect of the patella [5,6]. Recreating native anatomy is the core concept of MPFL reconstruction [5]. However, the femoral tunnel placement during MPFL reconstruction remains an unsettled technical issue [7,8]. It was reported that femoral tunnel malposition was the most common indication for revision surgery, and 32% complications after MPFL reconstruction were related to improper femoral tunnel placement [9,10].

Generally, visual–palpatory anatomic landmarks (i.e., the AT and the ME) [8,11] and fluoroscopy-guided radiographic landmarks (i.e., Schöttle’s point and Stephen’s point) [12,13,14,15] comprise the main approaches to intraoperative femoral tunnel placement during MPFL reconstruction. However, the limitations of both approaches have been investigated [16,17,18,19,20]. The accuracy of isolated palpatory determination varies from 52% to 72%, regardless of surgical experiences [14,21,22]. Positioning with fluoroscopy requires a true lateral radiograph with perfect overlap of both the condyle and the dorsal femoral cortex, which is challenging to obtain intraoperatively [17]. In patients with high-grade trochlear dysplasia, the difficulty is amplified due to aberrant trochlear morphology [18]. A deviation as small as 5° leads to a misinterpretation of 5 mm from the native femoral insertion, which can alter the isometry of the graft [16,17]. Therefore, methods of intraoperative femoral tunnel placement are still evolving.

The purpose of this technical note is to describe a novel approach to arthroscopic femoral tunnel placement during MPFL reconstruction for recurrent patellar dislocation. This technique provides a promising solution to femoral tunnel positioning based on arthroscopically visualized anatomic landmarks.

## 2. Surgical Techniques

### 2.1. Preoperative Evaluation

**Figure 1:**
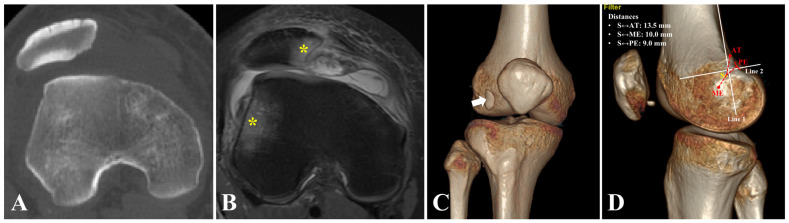
Preoperative imaging evaluation. Right knee. (**A**) Axial CT scan showing lateral patellar translation. (**B**) Magnetic resonance imaging showing kissing contusion of the medial patella and the lateral femoral condyle (yellow asterisks). (**C**) Anterior view of three-dimensional CT reconstruction showing an intra-articular loose body (arrow). (**D**) Medial view of three-dimensional CT reconstruction showing measurements between the ideal femoral attachment of the MPFL and three anatomic landmarks. The ideal position is assessed with Schöttle’s point (yellow spot). Line 1 is the extension of the posterior cortical line. Line 2 is the line perpendicular to line 1 intersecting the posterior origin of the medial femoral condyle. Schöttle’s point is located 1.3 mm anterior to line 1 and 2.5 mm distal to line 2. Preoperative measurements demonstrate that the ideal femoral tunnel lies 13.5 mm to the AT, 10.0 mm to the ME and 9.0 mm to the PE. AT, adductor tubercle. CT, computed tomography. ME, medial epicondyle. PE, posterior edge. S, Schöttle’s point.

The diagnosis of recurrent patellar dislocation is established with consideration of the patient’s history, a physical examination and diagnostic imaging. Patients often report repeated (at least two) events of lateral patellar dislocation. In outpatient clinic examination, a positive apprehension test suggests instability of the patellofemoral joint. Before surgery, computed tomography (CT) is routinely performed to evaluate the patellofemoral congruence (Figure 1A). Axial images with 1.0 mm slices are obtained and saved as Digital Imaging and Communications in Medicine data. Three-dimensional (3D) high-resolution bone surface rendering images are reconstructed on an AW Volume Share 4 workstation monitor (GE HealthCare, Fairfield, CT, USA). Magnetic resonance imaging is recommended to assess the injury pattern of the MPFL, concomitant ligament injury, chondral lesion and bone contusion (Figure 1B). The AT, the ME and the posterior edge (PE) are identified on 3D-CT. With measurement tools embedded in the workstation, the distances between the femoral attachment of the MPFL and the three bony landmarks are evaluated (Figure 1C,D).

### 2.2. Anesthesia and Patient Positioning

The patient is placed supine on a standard operating table. Subarachnoid anesthesia is administered. The head and body prominences are padded. The lower extremity is prepped and draped in the usual sterile fashion. A sterile pneumatic tourniquet is applied on the ipsilateral thigh.

### 2.3. Autograft Preparation

**Figure 2:**
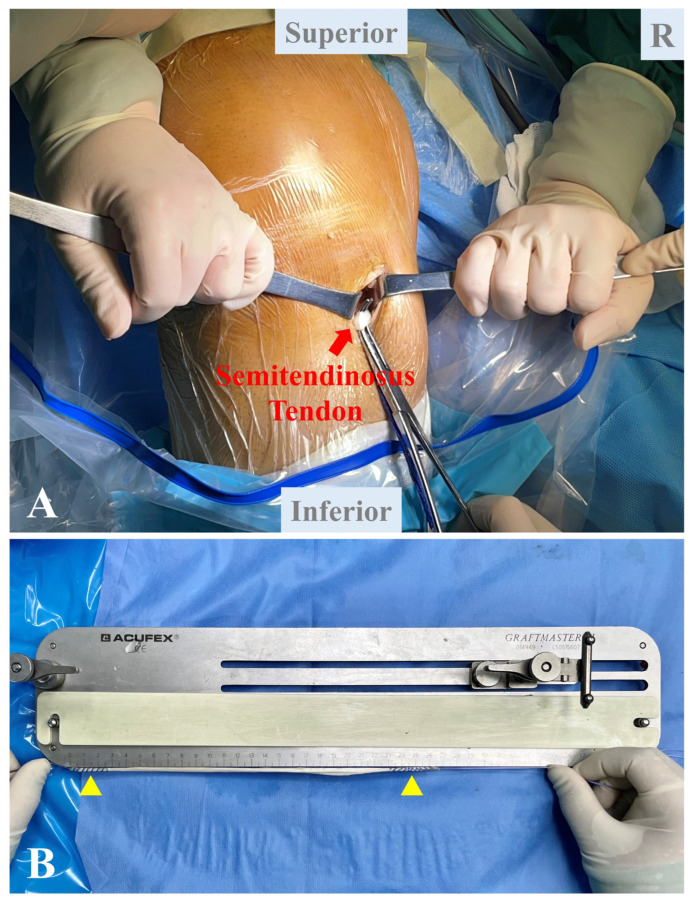
Autograft preparation. (**A**) The semitendinosus tendon is harvested through a 2 cm longitudinal incision 2 cm medial to the tibial tubercle. (**B**) The tendon is cleaned, and its two free ends are whip-stitched (yellow triangles).

On the ipsilateral knee, a 2 cm longitudinal incision is made approximately 2 cm medial to the tibial tubercle (Figure 2A). The semitendinosus tendon is identified and harvested using a closed tendon stripper. The tendon is cleaned, and its two free ends are whip-stitched with Orthcord sutures (DePuy Synthes) in standard baseball running fashion (Figure 2B). Generally, the autograft is between 22 and 28 cm in length and 6 mm in diameter.

### 2.4. Arthroscopic Portal Placement and Diagnostic Arthroscopy

**Figure 3:**
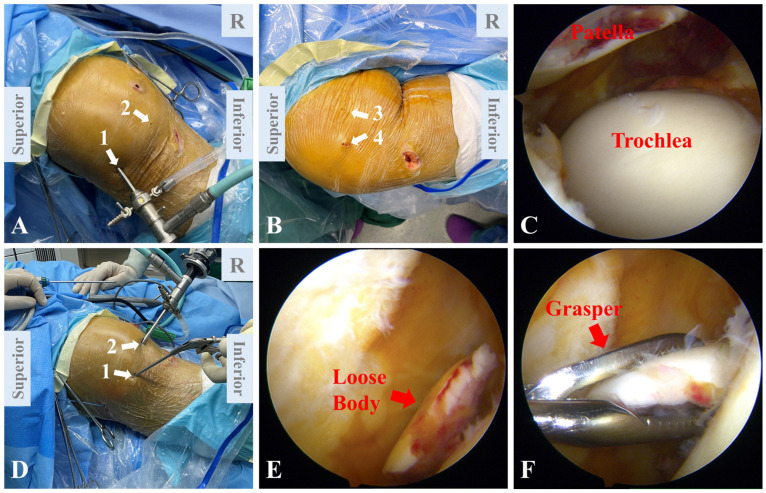
Arthroscopic portal placement and diagnostic arthroscopy. (**A**) An anterolateral portal (arrow 1) is made 1 cm lateral to the patellar tendon near the inferior pole of the patella. An anteromedial portal (arrow 2) is established 1 cm medial to the patellar tendon near the inferior pole. (**B**) Auxiliary medial portal 1 (AMP1, arrow 3) is made between the adductor tubercle and the medial epicondyle for the establishment of the femoral tunnel. Between the upper-third point of the medial patella and the AMP1, auxiliary medial portal 2 (AMP2, arrow 4) is created for the establishment of the patellar tunnel. (**C**) Diagnostic arthroscopy showing a flat trochlear groove and the patella lying laterally to the trochlea. (**D**) Removal of loose body is performed. The arthroscope is placed through the anteromedial portal (arrow 2), and a grasper is placed through the anterolateral portal (arrow 1). (**E**) Arthroscopic view of the loose body. (**F**) Arthroscopic view of the grasper removing the loose body.

In total, four arthroscopic portals are utilized in this technique (Figure 3A,B). An anterolateral portal is first established. A longitudinal incision is made approximately 1 cm lateral to the patellar tendon near the inferior pole of the patella, and the joint is then entered with a blunt trocar and a scope sheath. An anteromedial portal is created 1 cm medial to the patellar tendon near the inferior pole of the patella in a similar fashion. After palpation of the AT and the ME, a longitudinal incision is made between the two landmarks. Through the incision, auxiliary medial portal 1 (AMP1) is established. The upper-third point of the medial patella is identified through palpation. Between this point and the AMP1, auxiliary medial portal 2 (AMP2) is created.

Through the anterolateral portal, a diagnostic arthroscopy (30° 4.0 mm) is performed to assess patellofemoral congruence, loose body, concomitant ligament injury, meniscus injury and other intra-articular derangements (Figure 3C). The degree of chondral lesion in each compartment is assessed. Removal of loose bodies and chondroplasty are selectively performed depending on the results of arthroscopic inspection (Figure 3D–F).

### 2.5. Positioning of Femoral Tunnel

**Figure 4:**
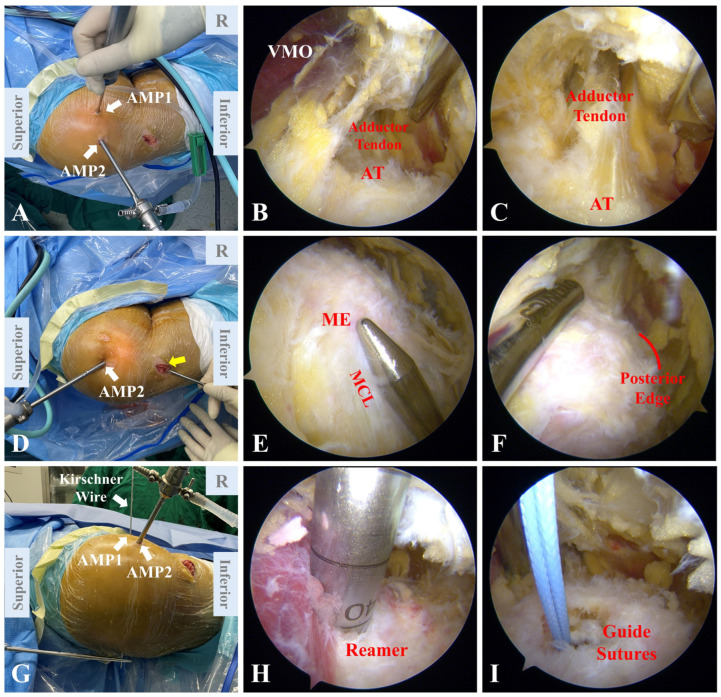
Positioning of femoral tunnel. (**A**) The arthroscope is placed through the AMP2 and a shaver is placed through the AMP1. (**B**) Under arthroscope, the VMO is first localized, followed by identification of the adductor tendon that lies flat against the distal part of the VMO. (**C**) The bony process where the adductor tendon attaches to the femur is identified as the AT. (**D**) The arthroscope is placed through the AMP2, and a blunt obturator is introduced through the incision for graft harvest (yellow arrow). (**E**) The blunt obturator is placed along the MCL arthroscopically, with its tip indicating the femoral attachment of the MCL, namely the ME. (**F**) The posterior edge is clearly exposed. (**G**) After the position of the femoral tunnel is confirmed, a Kirschner wire is introduced through the AMP1 and penetrates the femoral condyle. (**H**) A reamer is used to drill the femoral tunnel. (**I**) Guide sutures are passed through the tunnel. AMP, auxiliary medial portal. AT, adductor tubercle. MCL, medial collateral ligament. ME, medial epicondyle. VMO, vastus medialis oblique.

**Figure 5:**
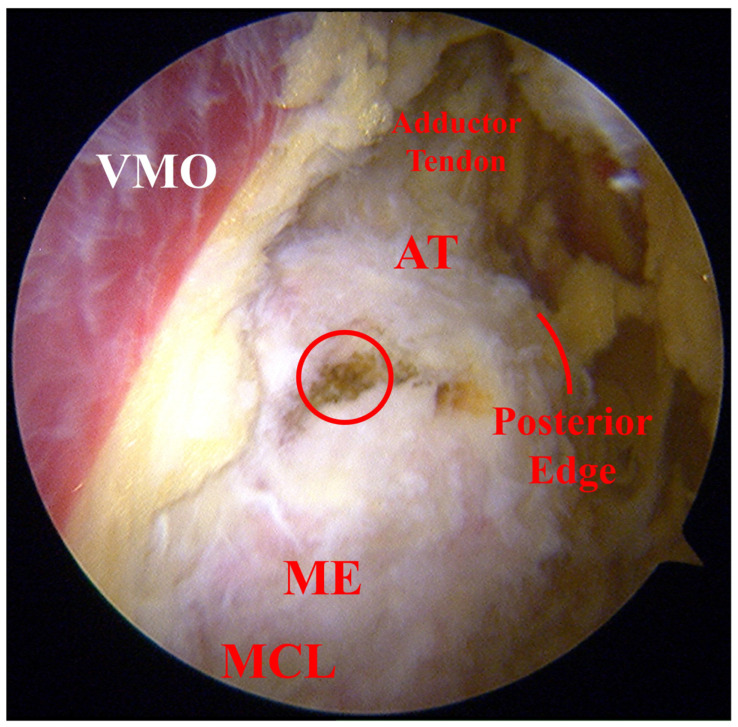
The relative position between the femoral tunnel (red circle) marked with cauterization and main anatomic landmarks under arthroscope. AT, adductor tubercle. MCL, medial collateral ligament. ME, medial epicondyle. VMO, vastus medialis oblique.

The arthroscope is placed through the AMP1. Through the AMP2, an arthroscopic 4.5 mm full radius shaver (Smith and Nephew) is used to debride along the femoral origin of the MPFL (Figure 4A). First, the vastus medialis oblique (VMO) is identified under arthroscope. The adductor tendon lies flat against the distal part of the VMO (Figure 4B). Along the adductor tendon, its femoral attachment is easily identified as the AT (Figure 4C). A blunt obturator is introduced through the incision used for graft harvest (Figure 4D). The blunt obturator is placed along the medial collateral ligament (MCL) under arthroscope. The tip of the blunt obturator indicates the femoral attachment of the MCL, namely the ME (Figure 4E). The shaver is used to clearly expose the PE, which is defined as the edge of the posteromedial cortex in the transition area between the medial condyle and the femoral shaft (Figure 4F) [8]. The femoral tunnel is placed on the midline between the AT and the ME. A probe with a scale is introduced into the joint to semi-quantitively evaluate the midpoint between the AT and the ME. The position of the femoral tunnel is marked using electrical cauterization (Figure 5). Taking a measurement on preoperative 3D-CT as a reference, a probe with a scale is used to evaluate the arthroscopic distance between the preliminary marker and the PE. This is to further ensure that the femoral tunnel is not localized too far back. The lower extremity is placed under lateral rotation to ensure that the transverse axial of the femoral condyle is perpendicular to the operating table. Once the proper position is settled, a 2.0 mm Kirschner wire is passed through the femoral condyle while keeping it perpendicular to the operating table (Figure 4G). A reamer is used to drill the femoral tunnel according to the diameter of the autograft (7 mm in general). Then, the medial part of the tunnel is over-reamed (Figure 4H). An eyelet pin with guide sutures is passed through the femoral tunnel (Figure 4I). Video demonstration of arthroscopic femoral tunnel positioning is presented in Supplementary Materials.

### 2.6. Positioning of Patellar Tunnel

**Figure 6:**
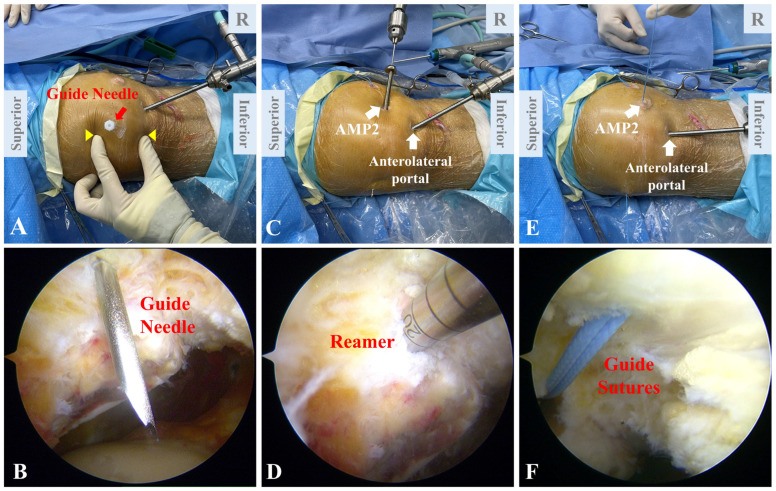
Positioning of patellar tunnel. (**A**) The superior pole, inferior pole (yellow triangles) and the medial margin of the patella are identified through palpation. A guide needle (red arrow) is inserted into the knee joint at the upper-third point of the medial patellar margin. (**B**) Arthroscopic view of the guide needle. (**C**) The arthroscope is placed through the anterolateral portal. A Kirschner wire is introduced through the AMP2 and penetrates the patella. (**D**) A reamer is used to drill the patellar tunnel. (**E**) Guide sutures are passed through the tunnel. (**F**) Arthroscopic view of the guide sutures. AMP, auxiliary medial portal.

The arthroscope is placed through the anteromedial portal (Figure 6A). The superior pole, inferior pole and the medial margin of the patella are identified through palpation. A guide needle is inserted into the knee joint to mark the upper-third point of the medial patellar margin (Figure 6B). The lower extremity is placed under lateral rotation to make sure the transverse axial of the patella is perpendicular to the operating table. Through the AMP2, a 2.0 mm Kirschner wire is introduced through the patella near the cartilage edge. The Kirschner wire is passed from the upper-third point marked by the needle to the anterior surface of the patella (Figure 6C). The patellar tunnel is then drilled using a reamer according to the diameter of the autograft (4.5 mm in general) (Figure 6D). An eyelet pin with guide sutures is passed through the patellar tunnel (Figure 6E,F).

### 2.7. Autograft Fixation

**Figure 7:**
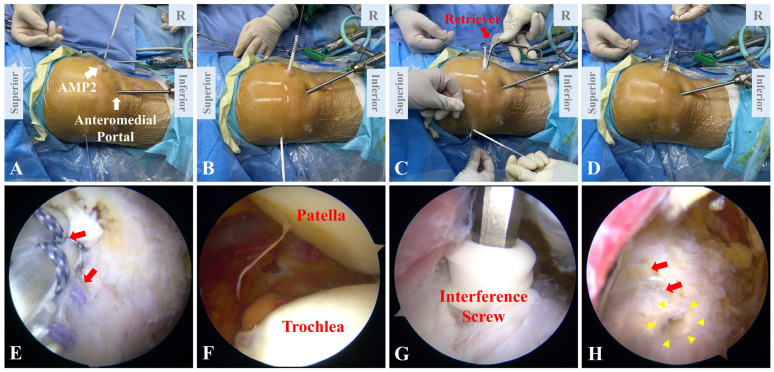
Autograft fixation. (**A**,**B**) Through the AMP2, the autograft is introduced into the patellar tunnel by guide sutures. (**C**) A retriever is introduced through the AMP2 to grasp the free end of the autograft. (**D**) The two ends of the autograft are pulled out through the AMP2, forming a double-bundle structure. (**E**) The double-bundle autograft (red arrows) is inserted into the femoral tunnel along the guide sutures. (**F**) Arthroscopic inspection demonstrates restored patellofemoral congruence. (**G**) A bioabsorbable interference screw is used to secure the femoral fixation. (**H**) The double-bundle autograft (red arrows) is fixed in the femoral tunnel with an interference screw (yellow triangles). AMP, auxiliary medial portal.

The autograft, along with the guide sutures, is passed through the patellar tunnel (Figure 7A,B). A retriever is introduced through the AMP2 to grasp the free end of the autograft and pull it back to the medial side, forming a double-bundle loop (Figure 7C,D). The retriever is then introduced through the AMP1 to grasp the two ends of the autograft, which are subsequently drawn into the femoral tunnel along the guide sutures (Figure 7E). The graft is first tensioned to the greatest extent. Then, the knee is cycled several times from full flexion to full extension until the graft remains isometric. This procedure is aimed at pre-conditioning of the autograft, which ensures the isometry and proper tension after fixation. Under arthroscope, patellofemoral alignment and patellar tracking are reassessed (Figure 7F). In this way, the tension of the graft is finally settled. With the knee flexed to 30 to 60 degrees, the two ends of the graft are fixed in the femoral tunnel using a 7 × 25 mm bioabsorbable interference screw (Smith and Nephew) (Figure 7G,H).

### 2.8. Postoperative Rehabilitation and Evaluation

**Figure 8:**
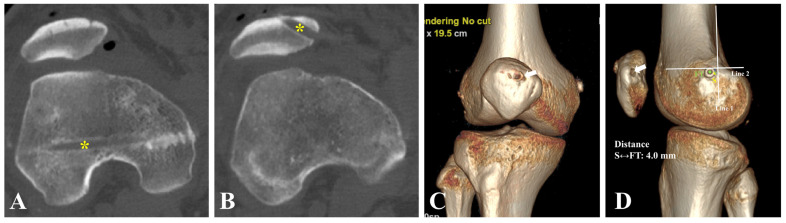
Postoperative imaging evaluation. Right knee. (**A**,**B**) Axial CT showing the femoral tunnel and the patellar tunnel (yellow asterisks). (**C**) Anterior view of three-dimensional CT reconstruction showing the patellar tunnel (white arrow). (**D**) Medial view of three-dimensional CT reconstruction. The patellar tunnel (white arrow) is located at the upper-third point of the medial patellar margin. The center of the arthroscopically localized femoral tunnel (green spot) lies 4.0 mm anterior to Schöttle’s point (yellow spot), which can be defined as precise tunnel placement according to prior studies [7,12,24]. CT, computed tomography. FT, femoral tunnel. S, Schöttle’s point.

Postoperatively, a hinged knee brace is applied. Tolerable weightbearing and physical therapy with passive range of motion are allowed the day after surgery. The patient progresses to quadricep activation and strengthening with active range of motion. Active knee flexion of 90 degrees is expected during the first 4 weeks, and full range of motion is expected after 3 months. Controlled sports activities are restricted to a minimum of 4 months, and full return to sports is recommended after 6 months. Three-dimensional CT scans are obtained within one week after surgery to evaluate the femoral tunnel positioning (Figure 8) [8]. To assess the accuracy, Schöttle’s point is first identified as the reference point on 3D-CT according to a previously described method [8,23]. The distance between the center of the femoral tunnel and Schöttle’s point is measured. A distance of <5 mm is regarded as precise tunnel placement [7,12,24].

## 3. Discussion

As the primary medial patellar soft-tissue stabilizer, the MPFL contributes to approximately 50% of the total force resistant to lateral patellar displacement [25]. Over 90% of patellar dislocations are accompanied by a ruptured or deteriorated MPFL [2,26]. A series of studies have confirmed the clinical efficacy of MPFL reconstruction in treating recurrent patellar dislocation [3,27,28]. Anatomic MPFL reconstruction is crucial to re-establish graft isometry and function, predominantly for the femoral tunnel placement [29].

Multiple cadaveric studies have described the femoral attachment of the MPFL. Stephen et al. [15] observed that the femoral insertion was located equidistant along the line from the AT to the ME, at the base of the groove between the bony prominences. Fujino et al. [11] reported that the femoral insertion of the MPFL consistently lay approximately 10 mm distal to the AT on the long axis of the femur. Chen et al. [29] found that the femoral origin of the MPFL was located at approximately 12 mm from the AT to the ME and 6 mm perpendicular–posterior to the border connecting the apexes of the two landmarks. Recreating native anatomy remains the key issue of femoral tunnel placement during MPFL reconstruction.

At present, the palpatory method and fluoroscopy-guided method comprise the main approaches to intraoperative femoral tunnel placement. Although the validity of the isolated palpatory method has been questioned in several studies [14,19,20], Chen et al. [29] argued that the effectiveness of palpation might have been underestimated by identifying the saddle sulcus as a reliable landmark where the MPFL was anatomically attached. However, accurately identifying the landmarks reported in cadaveric and radiographic studies during actual surgeries remains a demanding task. Certain factors may increase the difficulty of precise placement using free-hand palpation, including personal variation of bony prominences and obesity. Moreover, previous studies emphasized that no significant difference was observed between surgeons of varying expertise concerning the accuracy of palpatory determination [19,20]. As an important alternative, radiographic landmarks guided by intraoperative fluoroscopy have drawn great attention, with Schöttle’s point and Stephen’s point being the most commonly investigated references [12,24,30]. Several studies concluded that radiographic landmarks could be used to accurately determine the femoral origin of the MPFL [24,30], while Sanchis-Alfonso et al. [31] reported only 36.7% and 25.5% overlapping of the anatomic tunnel area using Schöttle’s and Stephen’s methods, respectively. A possible reason for this discrepancy might be different interpretations of the true lateral radiograph, which is a requisite for precise fluoroscopic positioning. Some studies argued that a true lateral image is often difficult to obtain intraoperatively and that measurements in the operative suite are largely subjective [16,17]. Moreover, Izadpanah et al. [18] reported difficulties in obtaining true lateral radiographs, especially in patients with high-grade trochlear dysplasia. An aberrant trochlear morphology significantly affects achieving complete overlap of both the condyle and the dorsal femoral cortex. Overall, the previous literature highlighted the difficulty of reproducible anatomical femoral tunnel placement during MPFL reconstruction, and intraoperative tunnel positioning techniques have continued to evolve.

To the best of our knowledge, there have been few studies focusing on arthroscopic femoral tunnel placement independent of fluoroscopy [8,32]. Our technique of femoral tunnel positioning is based on anatomic landmarks with direct visualization under arthroscope (Table 1). The first highlight of the described technique is the sequential identification of reliable anatomic landmarks, including the VMO, the adductor tendon, the AT, the MCL and the ME. Unlike the free-hand palpatory method, bony prominences are identified in our technique through the ligaments to which they attach while being arthroscopically visualized, which highly improves the accuracy of localization. The described procedure especially suits obese patients, for whom palpatory methods are difficult to perform. The next technical point is to determine the relative position of the femoral tunnel taking the AT and the ME as main reference points. Corresponding to prior anatomical studies [12,15,29], the femoral tunnel is placed on the midline between the AT and the ME. Moreover, the distance between the femoral attachment and the PE is evaluated on 3D-CT before surgery and re-measured with a probe intraoperatively, providing a semi-quantified reference as secondary positioning. With no need for fluoroscopy, the X-ray exposure is minimalized, and the potential influence of patient positioning or trochlear dysplasia is avoided.

Although arthroscopic femoral tunnel placement requires appropriate design of portals and experiences in deciphering anatomic landmarks, this technique provides a promising alternative to current intraoperative positioning methods. Further studies will be needed to verify its accuracy and reproducibility.

## Figures and Tables

**Table 1:** Highlights and pitfalls of discussed technique.

Highlights	Pitfalls
Direct visualization of anatomic landmarks under arthroscope Reliable and easily identified landmarks Precise placement without X-ray exposure Individualized preoperative evaluation with three-dimensional computed tomography Minimally invasive procedures Especially suits obese patients, for whom palpatory femoral tunnel placement is difficult to perform	Improper portal placement may result in insufficient visualization under arthroscope Identification of anatomical landmarks under arthroscope has a relatively longer learning curve

## Supplementary Materials

The following supporting information can be downloaded at: https://www.mdpi.com/article/10.3390/jcm12020680/s1, Video S1: A novel technique of arthroscopic femoral tunnel placement during medial patellofemoral ligament reconstruction for recurrent patellar dislocation.
